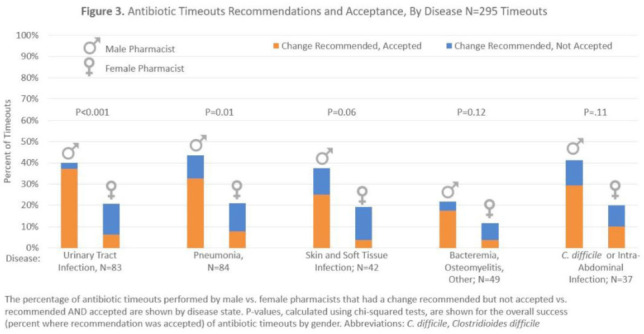# The effect of gender bias on acceptance of antibiotic stewardship recommendations by clinical pharmacists

**DOI:** 10.1017/ash.2022.188

**Published:** 2022-05-16

**Authors:** Valerie Vaughn, Daniel Giesler, Adamo Brancaccio, Daraoun Mashrah, Katie Sandison, Chaorong Wu, Jennifer Horowitz, Linda Bashaw, Adam Hersh

## Abstract

**Background:** Clinical pharmacists are a critical part of antibiotic stewardship. Stewardship often relies on relationships and persuasion, which may be affected by gender bias. Thus, we aimed to assess the association of sex with the acceptance of antibiotic stewardship recommendations. **Methods:** Between May and October 2019, medicine pharmacists at single hospital reviewed patients on antibiotics and–when a discharge was anticipated–led an antibiotic discussion (or “timeout”) prior to discharge. To explore differences in antibiotic timeout effectiveness by gender, we assessed the association of pharmacist sex with suggestion and acceptance of antibiotic changes using logistic regression controlling for patient characteristics. We also assessed whether hospitalist sex was associated with or moderated the effect of pharmacist sex on acceptance of timeout recommendations. **Results:** Between May 1, 2019, and October 31, 2019, pharmacists conducted 295 timeouts (patient characteristics in Fig. 1). Overall, 54% of timeouts were conducted by 12 female pharmacists and the remaining 46% were conducted by 8 male pharmacists. Overall, 82 (29%) of 295 timeouts resulted in a pharmacist recommending an antibiotic change, and male pharmacists were more likely to recommend a change: 52 (38%) of 137 versus 30 (19%) 158 (P **Conclusions:** In this discharge antibiotic intervention, timeouts conducted by women were less likely to result in an antibiotic change than those conducted by men. The difference in effectiveness resulted both from female pharmacists being less likely to recommend a change and from hospitalists being less likely to accept recommendations from a female pharmacist. These findings suggest that gender bias may play a role acceptance of antibiotic stewardship recommendations, which could affect antibiotic use, pharmacist job satisfaction, and patient outcomes.

**Funding:** None

**Disclosures:** None

**Figure:**
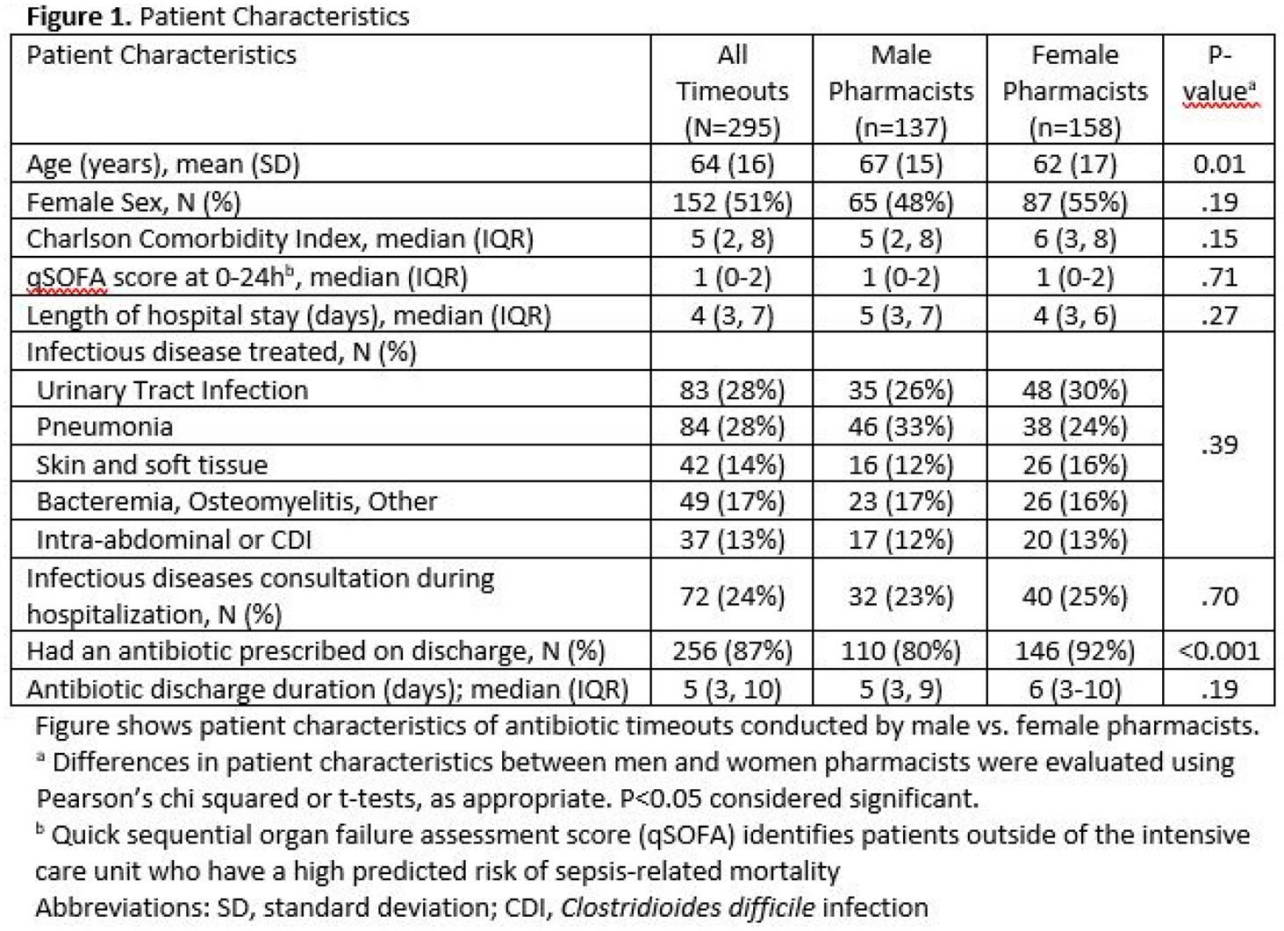

**Figure:**
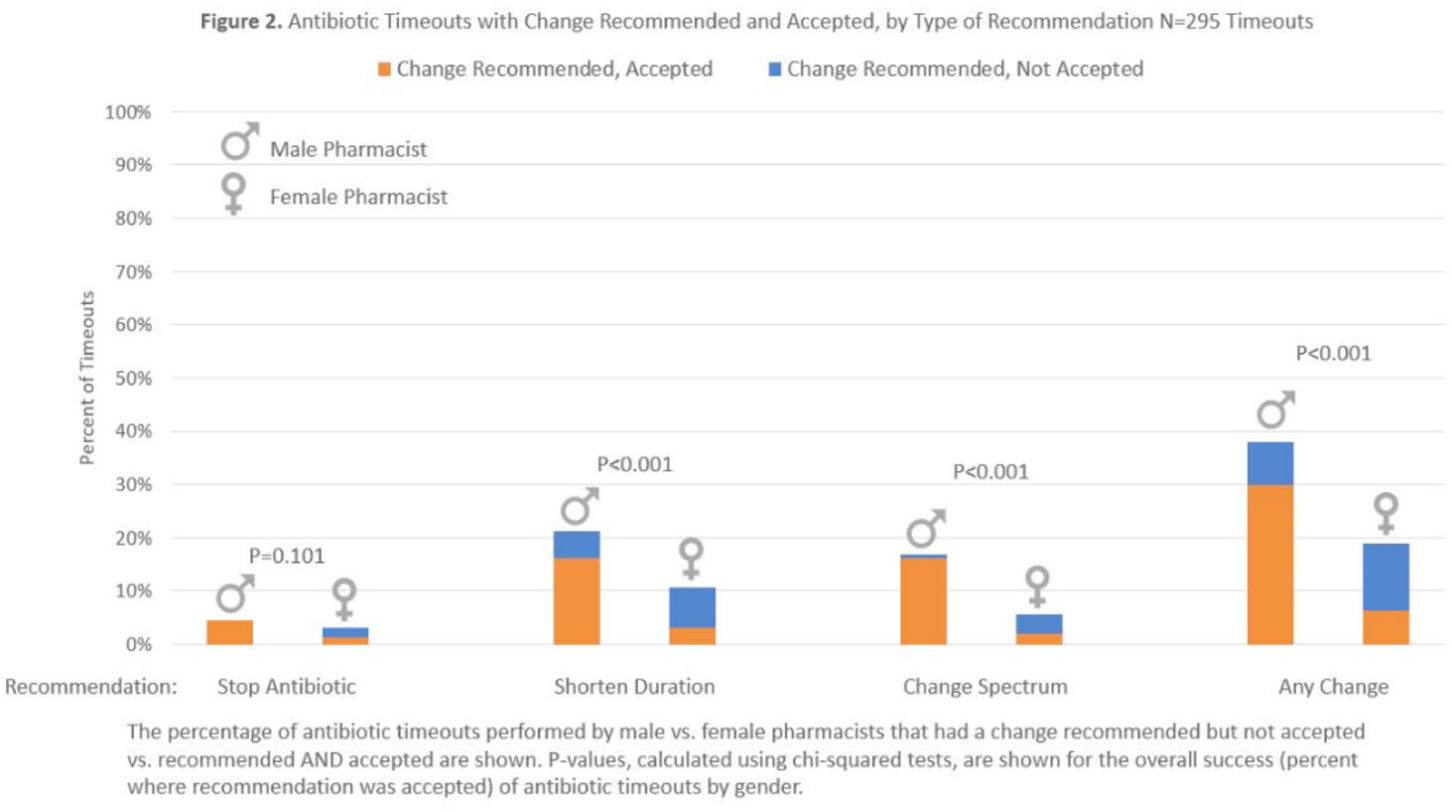

**Figure:**